# Precipitating factors of heart failure decompensation, short-term morbidity and mortality in patients attended in primary care

**DOI:** 10.1080/02813432.2020.1844387

**Published:** 2020-11-17

**Authors:** José María Verdu-Rotellar, Helene Vaillant-Roussel, Rosa Abellana, Lea Gril Jevsek, Radost Assenova, Djurdjica Kasuba Lazic, Peter Torsza, Liam George Glynn, Heidrun Lingner, Jacopo Demurtas, Beata Borgström, Sylvaine Gibot-Boeuf, Miguel Angel Muñoz

**Affiliations:** a Institut Català de la Salut, Barcelona, Spain; b Unitat de Suport a la Recerca de Barcelona, Fundació Institut Universitari per a la recerca a l'Atenció Primària de Salut Jordi Gol i Gurina (IDIAPJGol), Barcelona, Spain; c School of Medicine, Universitat Autònoma de Barcelona, Bellaterra, Spain; d Department of General Practice, Clermont Auvergne University, Faculty of Medicine, UPU ACCePPT, Clermont-Ferrand, France; e Departament de Fonaments Clinics, Facultat de Medicina, Universitat de Barcelona, Barcelona, Spain; f Medical Faculty, University of Maribor, Maribor, Slovenia; g School of Medicine, Medical University of Plovdiv, Plovdiv, Bulgaria; h Department of Family Medicine ‘Andrija Stampar’ School of Public Health, School of Medicine University of Zagreb, Zagreb, Croatia; i School of Medicine, Semmelweis University, Budapest, Hungary; j Health Research Institute and Graduate Entry Medical School, University of Limerick, Limerick, Ireland; k Hannover Medical School, Center for Public Health and Healthcare, Hannover, Germany; l Primary Care Department, Azienda Usl Toscana Sud Est, Grosseto, Italy; m Department of Clinical Sciences, Lund University, Malmö, Sweden

**Keywords:** Heart failure, decompensation, precipitating factors, primary care

## Abstract

**Objective:**

To evaluate the precipitating factors for heart failure decompensation in primary care and associations with short-term prognosis. *Design* Prospective cohort study with a 30-d follow-up from an index consultation. Regression models to determine independent factors associated with hospitalisation or death.

**Setting:**

Primary care in ten European countries. *Patients* Patients with diagnosis of heart failure attended in primary care for a heart failure decompensation (increase of dyspnoea, unexplained weight gain or peripheral oedema).

**Main outcome measures:**

Potential precipitating factors for decompensation of heart failure and their association with the event of hospitalisation or mortality 30 d after a decompensation.

**Results:**

Of 692 patients 54% were women, mean age 81 (standard deviation [SD] 8.9) years; mean left ventricular ejection fraction (LVEF) 55% (SD 12%). Most frequently identified heart failure precipitation factors were respiratory infections in 194 patients (28%), non-compliance of dietary recommendations in 184 (27%) and non-compliance with pharmacological treatment in 157 (23%). The two strongest precipitating factors to predict 30 d hospitalisation or death were respiratory infections (odds ratio [OR] 2.8, 95% confidence interval [CI] (2.4–3.4)) and atrial fibrillation (AF) > 110 beats/min (OR 2.2, CI 1.5–3.2). Multivariate analysis confirmed the association between the following variables and hospitalisation/death: In relation to precipitating factors: respiratory infection (OR 1.19, 95% CI 1.14–1.25) and AF with heart rate > 110 beats/min (OR 1.22, 95% CI 1.10–1.35); and regarding patient characteristics: New York Heart Association (NYHA) III or IV (OR 1.22, 95% CI 1.15–1.29); previous hospitalisation (OR 1.15, 95% CI 1.11–1.19); and LVEF < 40% (OR 1.14, 95% CI 1.09–1.19).

**Conclusions:**

In primary care, respiratory infections and rapid AF are the most important precipitating factors for hospitalisation and death within 30 d following an episode of heart failure decompensation.

**Key points**
- Hospitalisation due to heart failure decompensation represents the highest share of healthcare costs for this disease.
- So far, no primary care studies have analysed the relationship between precipitating factors and short term prognosis of heart failure decompensation episodes.
- We found that in 692 patients with heart failure decompensation in primary care, the respiratory infection and rapid atrial fibrillation (AF) increased the risk of short-term hospital admission or death.
- Patients with a hospital admission the previous year and a decompensation episode caused by respiratory infection were even more likely to be hospitalized or die within 30 d.

## Introduction

Heart failure is a chronic disease that affects approximately 1–2% of the population [1]. Its prevalence is increasing due to an aging population and therapeutic advances, among other factors [2]. The epidemiological dimension of heart failure, its clinical complexity and impact on patients’ quality of life all constitute a challenge for healthcare professionals. Moreover, the financial burden that it represents for a health system with limited resources makes this disease one of the greatest health, organisational and economic issues facing medical services globally [3]. Heart failure progression is characterised by increasingly frequent episodes of decompensation that often require hospitalisation [4], the greatest contributor to healthcare costs attributed to this disease [1,3]. There are a number of precipitating factors that can cause an episode of heart failure decompensation. The most frequent are respiratory infection, transgression in dietary recommendations, non-compliance with pharmacological treatment, hypertension and cardiac arrhythmias. Up to two thirds of these are avoidable, and timely identification could both improve disease management and reduce associated costs [5,6]. On average, heart failure patients attend their primary care centres more than 25 times a year, and their decompensations are often initially treated in these units [1]. In addition, primary care patients present greater comorbidity, the consultations and prescriptions generally including more than one pathology [7]. Most of those acutely admitted to hospital for common severe emergency diagnoses have been in contact with their family doctors during the month and year prior to admission [8]. All of the above, highlights the key role family doctors play in the care of such individuals. There is, however, a lack of information regarding precipitating factors in the prognosis of patients treated for heart failure decompensation in primary care. Evidence concerning the impact of such factors on decompensation episodes, mortality and hospital-admissions is largely based on data collected in hospital emergency rooms [9,10].

The objective of this study was, therefore, to identify the most frequent decompensation precipitating factors and analyse their association with short-term prognosis defined as hospitalisation/death within 30 d from an index consultation date.

## Methods

The HEFESTOS is an international prospective cohort study focused on identifying the precipitating factors involved in heart failure decompensation in primary care. In addition, it aims to create a predictive prognostic model in heart failure patients suffering from a decompensation episode.

The study was originally designed in Spain where a pilot study was carried out to test feasibility. Initially, 30 patients who had presented 13 events in one primary healthcare centre were recruited.

The protocol was presented in a meeting of the European General Practice Research Network where family doctors from nine countries agreed to collaborate. The European investigators then gathered in order to standardise the methodological procedures and extend the study to different countries. The study protocol was written in English with funding for translations to other languages.

Patients registered as having heart failure (according to the European Society of Cardiology criteria) [11] and attended in primary care centres, or managed by primary care physicians in home care, because of heart failure decompensation, were consecutively recruited in ten European countries. Recruitment was performed by 117 primary care physicians and nurses in Spain (72), France (12), Ireland (1), Germany (2), Italy (6), Slovenia (4), Croatia (16), Bulgaria (8), Hungary (1) and Sweden (3). Heart failure decompensation was considered as such when the patient presented any of the following: increase of dyspnoea, unexplained weight gain or appearance/increase of peripheral oedema.

Data on the following were collected: age, sex and comorbidity including hypertension, diabetes mellitus, hypercholesterolemia, ischemic heart disease, permanent atrial fibrillation (AF), chronic renal failure and stroke. New York Heart Association (NYHA) functional class, time from commencement of symptoms until primary care consultation, hospitalisation due to heart failure in the year prior to decompensation, left ventricular ejection fraction (LVEF) and precipitating factors were also assessed.

The following precipitating factors were taken into consideration if they appeared concomitantly with decompensation:

- Dietary transgression: if the patient reported liquid intake of more than 2.5 l a day and/or added salt to the food.
- Respiratory infection. Diagnosis was made by clinical exploration and confirmed in the medical records.
- Non-compliance with pharmacological treatment (self-reported).
- AF with a frequency > 110 beats per minute (bpm) requiring treatment or emergency referral.
- Other infections: signs and symptoms of infections other than respiratory ones.
- Cardiac ischemia: clinical and electrocardiographic signs of ischaemia that required changes in treatment or emergency referral.
- Intake of drugs associated with heart failure decompensation: non-steroidal anti-inflammatory drugs, effervescent tablets, corticosteroids, tricyclic antidepressants.
- Other precipitants: anaemia, worsening of renal function, hypertensive crisis, changes in diuretic treatment.

The primary outcomes were hospitalisation due to cardiovascular causes and/or mortality from any cause in the 30 d following the index consultation date. They were verified through consulting medical records and/or by phone.

### Statistical analyses

Categorical data are expressed using frequencies and percentages, while continuous data are expressed using means and standard deviations (SD). A chi-square test was used to compare data when it was categorical. A Student’s t-test was used for independent groups. Binary logistic regression analyses were used to investigate the effect of the characteristics of patients and precipitating factors in the primary outcome. Variables significantly (*p* < .05) associated with the primary outcome in the bivariate analysis were included as potential covariates in logistic regression models. Analyses were performed using R project version 3.6.0 (R Foundation for Statistical Computing, Vienna, Austria) [12].

## Results

Between August 2015 and December 2018, a total of 692 patients from ten countries were included. Women represented 55% of the participants and mean age was 81.2 (SD 8.90) years. Mean LVEF was 55% (SD 12%). The average time between consultation and onset of symptoms was 9.1 d (SD 9.1).

Among the 692 patients evaluated, 195 (28%) were hospitalised and 24 (3.5%) died during the first month following the decompensation episode. Table 1 describes the characteristics of patients according to the presence/absence of primary outcome.

**Table 1.** Baseline characteristics in the study population.

	Total *N* = 692	No hospitalisation/death at 30 d *N* = 492	Hospitalisation/death at 30 d *N* = 200	*p* Value
	*N* (%)	*N* (%)	*N* (%)	
Sex:				
Men	315 (45.5)	221 (44.9)	94 (47.0)	Ref.
Women	377 (54.5)	271 (55.1)	106 (53.0)	.437
Age, years^a^	81.2 (8.9)	81.2 (8.92)	81.2 (8.87)	.818
Body mass index, kg/m^2a^	30.3 (6.3)	30.3 (6.35)	30.5 (6.20)	.900
Diabetes mellitus	309 (44.7)	209 (42.6)	100 (50.0)	<.001
Cardiac ischaemia	263 (38.2)	187 (38.2)	76 (38.0)	.939
Chronic or paroxysmal atrial fibrillation	387 (56.1)	273 (55.7)	114 (57.0)	.821
Stroke	96 (13.9)	71 (14.5)	25 (12.5)	.296
Chronic renal disease	292 (42.3)	201 (41.0)	91 (45.5)	.148
Smoker	68 (9.90)	51 (10.5)	17 (8.50)	.147
Chronic obstructive pulmonary disease	204 (29.6)	135 (27.6)	69 (34.5)	.013
Hypertension	584 (88.8)	413 (88.2)	171 (90.0)	.056
Dyslipidaemia	373 (56.9)	255 (54.7)	118 (62.1)	<.001
NHYA^b^				<.001
I or II	188 (27.2)	163 (33.3)	25 (12.5)	–
III or IV	502 (72.8)	327 (66.7)	175 (87.5)	–
Hospital admission last year due to heart failure	175 (25.4)	105 (21.4)	70 (35.4)	<.001
Ejection fraction				
> = 50%	376 (73.4)	277 (77.2)	99 (64.7)	Ref.
40–49%	58 (11.3)	38 (10.6)	20 (13.1)	.109
< = 39%	78 (15.2)	44 (12.3)	34 (22.2)	.000
unknown	180 (26.0)	133 (27.0)	47 (23.5)	.154
ACE inhibitor/ARB	513 (74.9)	351 (71.9)	162 (82.2)	<.001
Betablockers	469 (68.4)	325 (66.5)	144 (73.1)	.215
Aldosterone antagonist	156 (22.9)	103 (21.2)	53 (27.0)	<.001
Loop diuretics	499 (73.0)	345 (71.0)	154 (77.8)	.068

^a^Mean (standard deviation). ^b^NHYA: New York Heart Association functional class.

At least one precipitating factor was identified in 156 (78%) of the 200 events, only one in 84 events (42%), two in 45 events (22%) and three or more in 27 events (14%). The most frequently identified precipitating factors were: respiratory infection (28%), transgression of dietary recommendations (27%) and non-compliance with pharmacological treatment (23%). Table 2 describes the precipitating according to the presence/absence of primary outcome.

**Table 2.** Precipitating factors in the study population.

	Total *N* = 692	No hospitalisation/death at 30 d *N* = 492	Hospitalisation/death at 30 d *N* = 200	*p* Value
	*N* (%)	*N* (%)	*N* (%)	
Respiratory infection one week before	194 (28.2)	106 (21.7)	88 (44.0)	<.001
Non-compliance with drug treatment	157 (22.8)	116 (23.7)	41 (20.7)	.261
Use of NSAID^a^, corticosteroids and tricyclic antidepressants	85 (12.4)	64 (13.1)	21 (10.6)	.524
Other infection one week before	49 (7.1)	31 (6.3)	18 (9.0)	.234
Atrial fibrillation at rate > 110 bpm	100 (14.5)	58 (11.8)	42 (21.0)	<.001
Non-compliance with diet^b^	184 (26.8)	148 (30.3)	36 (18.2)	<.001
Other arrhythmias	22 (3.1)	16 (3.2)	6 (3.0)	.984
Cardiac ischaemia	37 (5.3)	22 (4.4)	15 (7.5 )	.280
Anaemia	40 (5.8)	26 (5.3)	14 (7.0)	.380

^a^NSAID: non-steroidal anti-inflammatory drugs.

^b^Liquid intake of more than 2.5 l a day and/or added salt to the food.

In univariate analysis, patients suffering from a respiratory infection, or AF with a heart rate >110 beats/min, had a significantly higher probability of hospitalisation/death (OR 2.84, 95% CI 2.39–3.38) and (OR 2.17, 95% CI 1.49–3.16), respectively. In contrast, patients with dietary transgression had a lower probability of hospitalisation/death (OR 0.51, 95% CI 0.36–0.74). Regardless of the characteristics of patients; patients hospitalised during the previous year, presenting a history of diabetes mellitus, chronic obstructive pulmonary disease (COPD), hypercholesterolemia, LVEF < 40% or functional stage NYHA III or IV, treatment with ACE/or aldosterone antagonist had a higher probability of hospitalisation/death in the first 30 d following the episode (Table 3).

**Table 3.** Patient characteristics and precipitating factors predicting hospitalisation/death at 30 d in heart failure patients after an episode of decompensation.

		Hospitalisation/death at 30 d Univariate odds ratio [95% confidence interval]	Hospitalisation/death at 30 d Multivariate odds ratio [95% confidence interval]
	Total *N* = 692 *N* (%)		
Patient characteristics			
Sex:			
Men	315 (45.5)	1 (Ref.)	1 (Ref.)
Women	377 (54.5)	0.91 [0.70; 1.16]	0.99 [0.96; 1.03]
Age, years^a^	81.2 (8.9)	1.00 [0.98; 1.01]	0.99 [0.99; 1.00]
No diabetes mellitus	383 (55.3)	1 (Ref.)	
Diabetes mellitus	309 (44.7)	1.32 [1.14; 1.54]	
No chronic obstructive pulmonary disease	488 (70.4)	1 (Ref.)	
Chronic obstructive pulmonary disease	204 (29.6)	1.37 [1.07; 1.74]	
No dyslipidaemia	319 (43.1)	1 (Ref.)	
Dyslipidaemia	373 (56.9)	1.38 [1.21; 1.58]	
NHYA:^b^			
I or II	188 (27.2)	1 (Ref.)	1 (Ref.)
III or IV	502 (72.8)	3.49 [2.36; 5.14]	1.22 [1.14; 1.29]
No hospital admission last year due to heart failure	517 (74.6)	1 (Ref.)	1 (Ref.)
Hospital admission last year due to heart failure	175 (25.4)	2.13 [1.76; 2.57]	1.15 [1.11; 1.19]
Ejection fraction			
> = 50%	376 (73.4)	1 (Ref.)	1 (Ref.)
40–49%	58 (11.3)	1.46 [0.92; 2.31]	1.06 [0.98; 1.15]
< = 39%	78 (15.2)	2.14 [1.73; 2.65]	1.14 [1.09; 1.20]
Unknown	180 (26.0)	1.29 [0.91; 1.84]	1.06 [0.99; 1.14]
No ACE inhibitor/ARA2	179 (25.1)	1 (Ref.)	
ACE inhibitor/ARA2	513 (74.9)	1.87 [1.50; 2.34]	
No aldosterone antagonist	179 (77.1)	1 (Ref.)	
Aldosterone antagonist	156 (22.9)	1.38 [1.18; 1,60]	
Precipitating factors			
No respiratory infection one week before	498 (71.8)	1 (Ref.)	1 (Ref.)
Respiratory infection one week before	194 (28.2)	2.84 [2.39; 3.38]	1.19 [1.14; 1.25]
No atrial fibrillation at rate > 110 bpm	592 (85.5)	1 (Ref.)	1 (Ref.)
Atrial fibrillation at rate > 110 bpm	100 (14.5)	2.17 [1.49; 3.16]	1.22 [1.09; 1.35]
Compliance diet	508 (73.2)	1 (Ref.)	1 (Ref.)
Non-compliance diet^c^	184 (26.8)	0.51 [0.36; 0.74]	0.90 [0.85; 0.96]

^a^Mean (standard deviation). ^b^NHYA: New York Heart Association functional class.

^c^Liquid intake of more than 2.5 l a day and/or added salt to the food.

A multivariate analysis including underlying patient characteristics confirmed the association between the following variables and hospitalisation/death. With respect to precipitating factors: respiratory infection (OR 1.19, 95% CI 1.14–1.25) and AF with heart rate >110 beats/min (OR 1.22, 95% CI 1.10–1.35); and in relation to patient characteristics: NYHA III or IV (OR 1.22, 95% CI 1.15–1.29); previous hospitalisation (OR 1.15, 95% CI 1.11–1.19); and LVEF < 40% (OR 1.14, 95% CI 1.09–1.19) (Table 3).

These variables, with and without hospitalisation for heart failure the year before the consultation, were analysed separately, including interactions in the multivariate model. It was observed that hospitalisation in the previous year due to heart failure interacted with respiratory infection and dietary transgression. The probability of hospitalisation/death from an episode precipitated by respiratory infection was much greater if there had been a previous hospitalisation (OR 7.20, 95% CI 5.61; 9.25) than if there had not (OR 2.10, 95% CI 1.75; 2.53). Dietary transgression reduced the probability of an adverse event, although only in those who had not been previously hospitalised (OR 0.40, 95% CI 0.28; 0.58) (Table 4).

**Table 4.** Predictors of hospitalisation or death at 30 d in heart failure patients after an episode of decompensation and heart failure hospital admission last year.

	Hospital admission last year due to heart failure	No hospital admission last year due to heart failure
	Hospitalisation/death at 30 d Multivariate odds ratio [95% confidence interval] odds ratio [95% confidence interval]	Hospitalisation/death at 30 d Multivariate odds ratio [95% confidence interval] odds ratio [95% confidence interval]
Sex:		
Men	Ref.	Ref.
Women	0.70 [0.49; 1.00]	1.07 [0.83; 1.36]
Age, years^a^	1.04 [1.01; 1.06]	0.99 [0.98; 1.00]
NHYA^b^		
I or II	Ref.	Ref.
III or IV	5.11 [2.80; 9.34]	2.83 [1.63; 4.93]
Ejection fraction		
> = 50%	Ref.	Ref.
40–49%	1.43 [0954; 2.15]	1.52 [0.84; 2.72]
< = 39%	2.19 [1.09; 4.42]	2.07 [1.84; 2.33]
unknown	1.15 [0.82; 1.63]	1.00 [0.55; 1.82]
Respiratory infection one week before	7.20 [5.61; 9.25]	2.10 [1.75; 2.53]
Non-compliance with diet^c^	0.78 [0.56; 1.10]	0.40 [0.28; 0.58]

^a^Mean (standard deviation). ^b^NHYA: New York Heart Association functional class.

^c^Liquid intake of more than 2.5 l a day and/or added salt to the food.

## Discussion

### Statement of principal findings

Among the 692 patients from ten European countries presenting an episode of heart failure decompensation in primary care, in more than three out of four cases at least one precipitating factor was identified, the most frequent being respiratory infection. In addition, respiratory infection and rapid AF were associated with a high probability hospitalisation/death within 30 d which occurred in 29% of the decompensation episodes.

### Strengths and limitations

A major strength of this study is its prospective cohort design and international perspective (patients were recruited from ten European countries). A selection bias, however, could have occurred since the more severely ill subjects might have been identified. Nevertheless, the primary care physicians responsible for recruitment were instructed to consecutively include any patient with decompensated heart failure. Due to the low percentage of patients who died we could not analyse hospitalisation and death separately, consequently the main outcome was a combination of both.

Some studies [13] have shown that in primary care there is up to a third of heart failure overdiagnosis. To avoid such an issue, researchers were urged to only include patients who strictly met the clinical and echocardiographic criteria of the current European guidelines, despite the fact that 26% presented unknown LVEF, a proportion very similar to other studies in primary care [13,14].

A major limitation is not having determined the natriuretic peptides due to their low availability in primary care; even though NT-proBNP has a prognostic value in heart failure patients with an ejection fraction ≥ 40%. Clinical use is, however, limited as a result of large SDs, many co-morbidities and greater age [15].

### Findings in relation to other studies

To the best of our knowledge, this is the first study to analyse the role of precipitating factors in heart failure decompensation in primary care. Compared to previous similar studies, there was a greater proportion of women, higher comorbidity and a larger percentage presenting preserved ejection fraction which we attribute to the higher average age of our population [13,14,16–23]. The ratio of ACE/ARB, betablockers and aldosterone-antagonist patients were similar to other studies carried out in primary care [13,14].

The percentage of patients with a precipitating factor is within the range described by other authors (between 60 and 98%) [16–20,22,23]. We found more than one precipitating factors in more than one third of cases, similar to Formiga et al. [17]. Given the current variability among publications [16], the identification of preventable precipitating factors should be systematically assessed, as recommended in the Standardised reporting criteria [24]. In our case, we evaluated all the precipitating factors recommended in this document, and we added those which seemed to be relevant from a primary care point of view, such as the use of detrimental drugs.

The role of respiratory infection in heart failure decompensation has also been identified mainly in studies undertaken in hospital emergency rooms and internal medicine units [9,10,17–19,25]. Infections can produce a tachycardia secondary to fever which may shorten diastole and produce an increase in oxygen demand, particularly in susceptible subjects, such as those with COPD [25]. An observation confirmed in our study where there was a higher number of events associated with respiratory infections.

We detected that the probability of hospitalisation/death after a respiratory infection was even greater when patients had been admitted to hospital in the previous year, which is consistent with other authors [1]. Such finding could be explained by the fact that following hospitalisation the previous functional level was not fully attained, and the patients’ frailty thus increased.

Dietary transgression has also been commonly described as a precipitating factor for heart failure decompensation [16]. Non-compliance is often associated with a lack of knowledge about daily intake of liquids and salt, as well as the unanticipated sodium content of certain foods and drugs. Real intake could be underestimated, given that its detection is usually based on information received from the patients or their relatives [20]. Appropriate questioning on salt intake by primary healthcare professionals might present an opportunity to initiate educational and preventive measures in this regard.

AF is also often related to heart failure decompensation [16,17,21]. Fast and irregular heart rate, loss of atrioventricular synchrony, and decrease in the contribution of atrial contraction to ventricular filling make AF a frequent trigger for decompensation [21]. In our study, AF was identified as a possible precipitating factor in 14% of cases, a percentage slightly lower than that described by other authors [17,21]. The proportion of patients with ischemic heart disease and hypertensive emergency that we observed in was also lower than in other work. This could be related to a selection bias associated with patients from hospital emergency rooms where a greater severity of illness is expected than in those attended in primary care.

In our study, we considered the use of non-indicated/contraindicated medications as a possible cause of decompensation, an issue which was not mentioned in the Standardised reporting criteria [24].

Only a few studies have analysed the role of precipitating factors in the prognosis of heart failure [9,10,22,23]. We observed a 30-d mortality rate of 3.5%, lower than figures from hospital emergency rooms and cardiology and internal medicine hospitalisation units, where it ranged from 6 to 15%. The same trend was found regarding hospitalisations, which in our case did not arrive at 30%. Nevertheless, patients treated for decompensation in emergency room services reached almost 70%, possibly because there are more hospitalisations than referrals or because those in the emergency rooms have a more severe condition.

Regarding comorbidity and decompensation short-term prognosis, in the univariate analysis the presence of diabetes mellitus and COPD was related to a greater probability of admission/death. This relationship however, was lost when performing the multivariate analysis. The presence of these two conditions has been related to the long-term prognosis of heart failure, particularly with ejection fraction [26], nevertheless, at short term other factors, such as precipitants may play a greater role.

One of the most striking findings of the study was that patients in whom dietary transgression had been identified had a lower probability of being hospitalised or dying. A lower probability that was not observed in those who had been hospitalised for heart failure in the previous year. A number of studies have reported that a dietary sodium restriction < 2 g/d provides benefits only in more advanced stages, and is neutral or even harmful in milder stages which could partly explain our results [27]. Another possibility might be that some patients could have been misdiagnosed as heart failure, and they did not follow dietary restrictive recommendations.

### Meaning of the study

In a primary care study from 10 European countries, we identified respiratory infections and rapid AF as the most important precipitating factors for hospitalisation and death within 30 d following an episode of heart failure decompensation.

Patients with a hospital admission the previous year, and a decompensation episode caused by respiratory infection, were even more likely to be hospitalised or die within 30 d.
